# Periprocedural and In-Hospital Outcomes Among Percutaneous Coronary Intervention in Saphenous Vein Graft: A Retrospective Observational Study at a Tertiary Care Hospital in South Asian Country

**DOI:** 10.7759/cureus.14251

**Published:** 2021-04-01

**Authors:** Abdul Baqi, Sheema Saadia

**Affiliations:** 1 Cardiology, Aga Khan University Hospital, Karachi, PAK; 2 Cardiology, Aga Khan University Hospital, Karachi, PAK

**Keywords:** saphenous vein graft, percutaneous coronary intervention, coronary artery bypass graft surgery, acute coronary syndrome, chronic stable angina

## Abstract

Background

Saphenous vein graft (SVG) may occlude either early or several months to years after coronary artery bypass graft (CABG) surgery. Doing re-do CABG surgery is associated with higher complication and mortality rate as compared to percutaneous coronary intervention (PCI) in SVG. However, PCI of SVG is associated with more periprocedural and in-hospital complications as compared to PCI of native coronary arteries. Due to the scarcity of local data in this regard, this study was designed to estimate the periprocedural and in-hospital outcomes among PCI in SVG.

Objectives

We aim to study the periprocedural and in-hospital outcomes among patients who underwent PCI in SVG.

Methods

It is a retrospective observational study. We reviewed hospital record files of 167 consecutive patients, admitted to Aga Khan University Hospital, Karachi, from January 2010 to December 2019, who underwent PCI in SVG.

Results

Out of 167 patients, 145 (86.8%) were male with a mean age of 72.26 (±8.46) years. Hypertension was the most common comorbid condition. Majority of 141(84.4%) patients presented within 6-10 years since the last CABG done. Seventy-eight (46.7%) patients presented with non-ST elevation myocardial infarction (NSTEMI). Patients who presented with acute coronary syndrome (ACS), 51 (36.9%), and 21 (15.2%) had congestive heart failure and cardiogenic shock on presentation respectively. Coronary angiography was performed in the majority of 155 (92.8%) patients through the femoral artery. The body of the SVG was the most common site affected by the disease. In 88 (52.7%) patients stents were deployed in SVG to obtuse marginal (OM). Drug-eluting stents (DES) were used in 124 (74.3%) patients. 22 (13.2%) of patients developed periprocedural complications, predominantly slow flow and 7 (4.2)% patients had in-hospital complications.

Conclusions

PCI of SVG is associated with a high procedural success rate and acceptable risk for periprocedural and in-hospital complications. PCI of SVG may be considered as a safe and efficacious option for the percutaneous intervention of SVG lesions.

## Introduction

Coronary artery disease (CAD) is one of the leading causes of death in South East Asia and the rest of the world [1]. Besides risk factors modification and optimal medical therapy, revascularization (percutaneous coronary intervention [PCI] and coronary artery bypass graft [CABG]) is one of the main streamlined part of the management protocol in patients with CAD [2].

CABG has been used successfully in patients with left main and severe three vessels CAD [3]. Occlusion of saphenous vein graft (SVG) may occur either early or after several months or years after CABG. Occlusion of graft is usually caused by graft failure or a combination of graft failure and progression of coronary atherosclerosis. The type of lesion responsible for graft occlusion depends on the time of onset of ischemic symptoms. Ischemia in acute graft occlusion is mostly due to surgical problems. Late graft occlusion is usually caused by disease progression, graft degeneration and new atherosclerosis [4].

Approximately 10% of SVGs are occluded early postoperatively, 20% at one year and 50% at 10 years of follow-up. Furthermore, 70% of SVGs were found to be diffusely diseased at 10 years [5]. The mode of intervention in patients presenting with graft occlusion remains a subject of debate. Doing re-do CABG surgery has higher complication rates and mortality as compared to PCI in SVG [6]. Re-do CABG is related to higher morbidity and mortality rates moreover as poorer outcomes as compared to initial CABG surgery [7].

PCI of SVG has a better outcome as compared to re-do CABG. But PCI of SVG is related to additional periprocedural and in-hospital complications as compared to PCI of native coronary arteries [8]. Due to scarcity of local data in this regard, this study was designed to estimate the periprocedural (dissection, perforation, slow flow/no flow, procedural failure and emergency CABG) and in-hospital outcomes (target lesion revascularization [TLR], major bleeding, Hematoma needed blood transfusion and mortality) among PCI in SVG.

## Materials and methods

Study population

It is a retrospective observational study done at Aga Khan University Hospital, Karachi, which is a multidisciplinary tertiary care hospital with a cardiology and cardiac surgery center and it is one of the main referral health facilities in Pakistan. This study was approved by ethical review committee. We reviewed hospital record files of consecutive 167 patients, admitted from January 2010 to December 2019, who underwent PCI in SVG.

For all patients, demographic information including age and gender, mode of presentation, cardiogenic shock status, and congestive heart failure were recorded after reviewing written medical records. Comorbidities were tabulated including Diabetes mellitus (DM), hypertension, prior CAD, prior PCI, chronic kidney disease (CKD), chronic obstructive pulmonary disease (COPD), smoking, peripheral arterial disease, cerebrovascular disease, ejection fraction (EF), and years since last CABG done.

Procedure

Before the procedure, all patients were loaded with oral aspirin (300 mg) and clopidogrel (300-600 mg). After informed consent for procedure, coronary angiography was performed by an interventional or non-interventional cardiologist. The vascular access site was the discretion of the primary operator. During the procedure, all patients received intravenous un-fractionated heparin according to body weight. Details of coronary angiography including vascular access site, graft anastomosed to the coronary artery, location of the lesion in SVG were recorded.

PCI in SVG was performed by an interventional cardiologist. The use of antiplatelet glycoprotein (GP) IIb/IIIa inhibitor and type of stent {DES or bare-metal stent (BMS)} was at the discretion of the operator. Details of PCI in SVG including stent characteristics (number, type, and size), direct stenting, thrombus aspiration, pre-dilation, post-dilation, size of balloon used for post-dilation, use of intravascular ultrasound (IVUS), fractional flow reserve (FFR), and GP IIb/IIIa inhibitor were recorded. Periprocedural outcomes related to PCI in SVG including dissection, perforation, slow flow/no flow, procedural failure, and emergency CABG were recorded.

All patients were followed from the time of the procedure to the time of discharge or death. Patients were observed during the hospital stay for PCI-related in-hospital complications including major bleeding, hematoma needed a blood transfusion, TLR, and in-hospital death.

Procedural success was defined as the achievement of residual in-stent stenosis of less than 30%, associated with thrombolysis in myocardial infarction (TIMI) flow grade 3, in the absence of a dissection more than D1 and perforation without the occurrence of death or repeat TLR during the index hospital stay [9]. All deaths were considered to be cardiac unless an unequivocal non-cardiac cause could be established [10]. TLR was defined as repeat PCI or CABG performed because of restenosis of the target lesion in association with angina or objective evidence of myocardial ischemia [11]. Major bleeding is defined as patients with intracranial hemorrhage or a ≥5 g/dl decrease in hemoglobin concentration or a ≥15 % absolute decrease in hematocrit [12].

Statistical analysis

To describe the characteristics of the study population we reported frequencies and proportions for the categorical variables such as gender, diabetes or hypertension, etc. We checked the normality assumption for continuous variables by histograms superimposed with the normal curve. We reported mean and standard deviation for normally distributed continuous variables (age), otherwise, we reported median and interquartile range (IQR) if data were skewed. We used a Chi-squared test or Fisher’s exact to assess the frequency distribution and the relationship between co-variates and periprocedural and in-hospital complications for categorical variables. We considered a p-value of less than 0.05 for significant results. All statistical analyses were performed using the Statistical Package for Social Sciences (SPSS) version 23 (IBM Corp., Armonk, NY).

## Results

Baseline characteristics

Overall, we analyzed data for 167 admitted patients. The mean age of the patients was 72.26 years with an SD of 8.46. We found that more than three-fourths of the patients 145 (86.6%) were males. Moreover, 113 (67.7%) of the patients were diabetic and 153 (91.6%) were hypertensive (Table 1). Around three-fourth 138 (82.6%) of the patients had dyslipidemia and 36 (21.6%) were found to be smokers. We did not find a high prevalence of COPD in the study participants, however, around one fourth 40 (24%) of patients were suffering from CKD, 120 (71.9%) of the patients had a history of prior MI, 59 (35.3%) had a history of prior PCI, 6 (3.6%) reported having peripheral arterial disease, and 13 (7.8%) of the patients had cerebrovascular disease. The duration since last CABG was 6 to 10 years for more than three-fourths 141 (84.4%) of the patients (Table 1).

**Table 1:** Patients demographics and baseline characteristics (n = 167). MI: myocardial infarction; CVA: cerebrovascular accident; PCI: percutaneous coronary intervention; CABG: coronary artery bypass graft; SD: standard deviation; CKD: chronic kidney disease; COPD: chronic obstructive pulmonary disease.

Variables	n (%)
Age, mean (SD) year	72.26 (8.46)
Male	145 (86.8)
Diabetes mellitus	113 (67.7)
Hypertension	153 (91.6)
Dyslipidemia	138 (82.6)
Smoking status	36 (21.6)
COPD	4 (2.4)
CKD	40 (24)
Prior MI	120 (71.9)
Prior PCI	59 (35.3)
Peripheral arterial disease	6 (3.6)
Cerebrovascular disease	13 (7.8)
Years since last CABG done	
0-1	3 (1.8)
2-5	23 (13.8)
6-10	141 (84.4)

Mode of presentation and hemodynamic status 

Majority of patients, presented with NSTEMI 78 (46.7%) followed by unstable angina 36 (21.6%). Only 24 (14.4%) patients presented with STEMI and 29 (17.4%) patients presented with chronic stable angina (CSA). Of patients who presented with ACS, 51 (36.9%) had congestive heart failure and 21 (15.2%) had a cardiogenic shock on presentation. The median EF of the patients was 45% with an IQR of 20 (Table 2).

**Table 2:** Mode of presentation and hemodynamic status. NSTEMI: non-ST elevation myocardial infarction; STEMI: ST-elevation myocardial infarction; EF: ejection fraction; IQR: interquartile range.

Variables	n (%)
Mode of presentation	
Chronic stable angina	29 (17.4)
Unstable angina	36 (21.6)
NSTEMI	78 (46.7)
STEMI	24 (14.4)
Congestive heart failure	51 (36.9)
Cardiogenic shock	21 (15.2)
EF% (median- IQR)	45 (20)

Vascular access and coronary angiography characteristics

Coronary angiography was performed in the majority of 155 (92.8%) patients through the femoral artery followed by left radial artery 8 (4.8%), and only in 4 (2.4%) patients, was performed through the right radial artery. In 79% of patients, LIMA was anastomosed to the left anterior descending artery (LAD). The anastomosis of SVG to OM, LAD, RCA, PDA, Ramus, and Diagonal was found in 152(91%), 35(21%), 77(46.1%), 65 (38.9%), and 20 (12%), 17 (10.2%) respectively. Only in 5 (3%) and 1 (0.6%) patients, RIMA and radial artery were anastomosed with coronary arteries respectively. Coronary angiography revealed the site of lesion in SVG, 105(62.9%) of the patients had a lesion in the body of SVG, 41 (24.6%) had lesion at the aortic anastomosis and 21 (12.6%) had lesion at the distal anastomosis (Table 3).

**Table 3:** Coronary angiography characteristics. SVG: saphenous vein graft; LAD: left anterior descending artery; OM: obtuse marginal; RCA: right coronary artery; PDA: posterior descending artery; LIMA: left internal mammary artery; RIMA: right internal mammary artery.

Variables	n (%)
Vascular access	
Femoral artery	155 (92.8)
Left radial	8 (4.8)
Right radial	4 (2.4)
Graft anastomosed to	
LIMA to LAD	132 (79)
SVG to LAD	35 (21)
SVG to diagonal	17 (10.2)
SVG to ramus	20 (12)
SVG to OM	152 (91)
SVG to RCA	77 (46.1)
SVG to PDA	65 (38.9)
Radial	1 (0.6)
RIMA	5 (3)
LIMA to diagonal	4 (2.4)
SVG lesion location	
Aortic anastomosis	41 (24.6)
Body	105 (62.9)
Distal anastomosis	21 (12.6)

PCI of SVG characteristics

The majority of stents were deployed in SVG to OM 88 (52.7%) followed by RCA 26 (15.6%). Ten (6%) patients had PCI of two grafts. Thrombus aspiration was performed in 45 (26.9%) of patients due to the high burden of thrombus. GP IIb/IIIa inhibitor was used in 65 (38.9%) patients. The lesion was pre-dilated in 103 (61.7%) of patients, while in 64 (38.3%) patients stents were directly deployed. DES was used in 124 (74.3%) patients, while BMS was the second most common which was used in 37 (22.2%) of patients, and in 6 (3.5%) patients only drug-eluting balloon (DEB) angioplasty was performed. In 112(67.1%) of patients only one stent was used and in 55 (32.9%) patients two or more stents were used. The stents used had a mean length and range of 31.24/8-144 mm. Post dilation was done in 124 (74.3%) patients with a mean balloon size of 3.36 mm and with a minimum to a maximum size of 2.0-6.0 mm. IVUS was performed before and after stent deployment in 12 (7.2%) patients (Table 4).

**Table 4:** PCI of SVG characteristics. DES: drug-eluting stent; BMS: bare-metal stent; DEB: drug-eluting balloon; POBA: plane old balloon angioplasty; IVUS: intravascular ultrasound; GP: glycoprotein; LAD: left anterior descending artery; OM: obtuse marginal; RCA: right coronary artery; PDA: posterior descending artery; IQR: interquartile range: PCI: percutaneous coronary intervention; SVG: saphenous vein graft.

Variables	n (%)
Saphenous vein graft stented	
SVG to LAD	12 (7.2)
SVG to Diagonal	17 (10.2)
SVG to Ramus	2 (1.2)
SVG to OM	88 (52.7)
SVG to RCA	26 (15.6)
SVG to PDA	22 (13.2)
Number of stents deployed in patients	
One	112 (67.1)
Two or more	55 (32.9)
Type of stents	
DES	124 (74.3)
BMS	37 (22.2)
POBA/DEB	6 (3.5)
Stent diameter (median- IQR)	3.5 (3)
Stent length mean with min-max (mm)	31.24/8-144
Direct stenting	64 (38.3%)
Pre-dilation	103 (61.7%)
Post-dilation	124(74.3%)
Thrombus aspiration	45 (26.9%)
Balloon size for post dilation mean/min-max (mm)	3.36/2.0-6.0
IVUS used	12 (7.2%)
GP IIb/IIIa inhibitor use	65 (38.9%)

Incidence of periprocedural and in-hospital complications

One of the main objectives of the study was to measure the incidence of overall periprocedural and in-hospital complications. The findings of the study illustrated that the incidence of periprocedural complications among the study population was 22 (13.2%), while the incidence of in-hospital complications was relatively lower 7 (4.2%) as shown in Table 5. Within periprocedural complications, the most common complication was slow flow with an incidence of 13 (7.8%) followed by dissection 8 (4.8%), and procedural failure 1 (0.6%) as shown in Table 5. Likewise, the most common type of in-hospital complication was mortality 4 (2.4%) followed by hematoma 3(1.8%) as shown in Table 5.

**Table 5:** Incidence of different types of periprocedural and in-hospital complications (n = 167). TLR: target lesion revascularization; CABG: coronary artery bypass graft.

Periprocedural complications	n	%	In-hospital complications	n	%
Overall periprocedural complications	22	13.2	Overall in-hospital complications	7	4.2
Dissection	8	4.8	Major bleeding	0	0
Perforation	0	0	Hematoma	3	1.8
Slow flow	13	7.8	TLR	0	0
Emergency CABG	0	0	Death	4	2.4
Procedural failure	1	0.6			

Characteristics of patients by type of complications

We found that 50% of the patients with periprocedural complications were older than 70 years when compared with 62.8% of the patients without periprocedural complications. Similarly, 63.6% of the patients with periprocedural complications and 68.3% of their counterparts were diabetic (p-value: 0.63). A significantly higher proportion of patients without periprocedural complications (93.8%) were hypertensive compared to patients with periprocedural complications (77.3%; p-value: 0.009). About one-third of the patients with periprocedural complications (31.8%) were smokers when compared to 20.0% of patients without periprocedural complications (p-value: 0.21) as shown in Table 6. Around 4.5% of the patients with periprocedural complications had CKD as opposed to 26.9% of the patients without periprocedural complications (p-value: 0.02). Around 9% of the patients with periprocedural complications had CSA when compared to 18.6% of patients without periprocedural complications. We did not find any significant differences between patients with and without periprocedural complications on factors such as gender, dyslipidemia, prior MI, cerebrovascular disease, and prior PCI as shown in Table 6.

**Table 6:** Demographic and clinical characteristics of patients by type of complications (n = 167). DM: diabetes mellitus; COPD: chronic obstructive pulmonary disease; CKD: chronic kidney disease; MI: myocardial infarction; PCI: percutaneous coronary intervention; PAD: peripheral arterial disease; CVA: cerebrovascular disease; CABG: coronary artery bypass graft; CSA: chronic stable angina; ACS: acute coronary syndrome.

	Periprocedural complications					In-hospital complications				
	No		Yes		p-value	No		Yes		p-value*
	n	%	n	%		n	%	n	%	
Age (years)										
≤ 70	54	37.2	11	50	0.25	63	39.4	2	28.6	0.70
>70	91	62.8	11	50		97	60.6	5	71.4	
Gender										
Male	126	86.9	19	86.4	0.94	140	87.5	5	71.4	0.23
Female	19	13.1	3	13.6		20	12.5	2	28.6	
DM										
No	46	31.7	8	36.4	0.63	51	31.9	3	42.9	0.41
Yes	99	68.3	14	63.6		109	68.1	4	57.1	
Hypertension										
No	9	6.2	5	22.7	0.009	13	8.1	1	14.3	0.46
Yes	136	93.8	17	77.3		147	91.9	6	85.7	
Dyslipidemia										
No	26	17.9	3	13.6	0.77	29	18.1	0	0	0.25
Yes	119	82.1	19	86.4		131	81.9	7	100	
Smoking status										
No	116	80	15	68.2	0.21	126	78.8	5	71.4	0.47
Yes	29	20	7	31.8		34	21.3	2	28.6	
COPD										
No	142	97.9	21	95.5	0.43	157	98.1	6	85.7	0.16
Yes	3	2.1	1	4.5		3	1.9	1	14.3	
CKD										
No	106	73.1	21	95.5	0.02	125	78.1	2	28.6	0.009
Yes	39	26.9	1	4.5		35	21.9	5	71.4	
Prior MI										
No	43	29.7	4	18.2	0.32	46	28.7	1	14.3	0.36
Yes	102	70.3	18	81.8		114	71.3	6	85.7	
Prior PCI										
No	4	64.8	14	63.6	0.91	102	63.7	6	85.7	0.22
Yes	51	35.2	8	36.4		58	36.3	1	14.3	
PAD										
No	139	95.9	22	100	1	155	96.9	6	85.7	0.23
Yes	6	4.1	0	0		5	3.1	1	14.3	
CVA										
No	134	92.4	20	90.9	0.68	148	92.5	6	85.7	0.44
Yes	11	7.6	2	9.1		12	7.5	1	14.3	
Years since CABG										
0-1 year	3	2.1	0	0	0.66	3	1.9	0	0	0.93
2-5 years	19	13.1	4	18.2		22	13.8	1	14.3	
6-10 years	123	84.8	18	81.8		135	84.4	6	85.7	
Mode of presentation										
CSA	27	18.6	2	9.1	0.27	29	18.1	0	0	0.26
ACS	118	81.4	20	90.9		131	81.9	7	100	

Likewise, we explored differences in the sociodemographic and clinical characteristics by in-hospital complications. The study findings revealed that 71.4 % of the patients with in-hospital complications were older than 70 years when compared with 60.6% of the patients without in-hospital complications. Similarly, 57.1% of the patients with in-hospital complications and 68.1% of their counterparts were diabetic (p-value: 0.41). Further, 85.7% of patients with in-hospital complications were hypertensive compared to 91.9% of the patients without in-hospital complications, however, unlike periprocedural complications the findings were not significant for hypertension (p-value: 0.46). About more than a quarter of the patients with in-hospital complications (28.6%) were smokers when compared to 21.3% of patients without in-hospital complications (p-value: 0.21) as shown in Table 6. Around 71.4% of the patients with in-hospital complications had CKD as opposed to 21.9% of the patients without in-hospital complications and findings were statistically significant (p-value: 0.009). We did not find any significant differences between patients with and without in-hospital complications on factors such as gender, dyslipidemia, prior MI, cerebrovascular disease, and prior PCI as shown in Table 6.

## Discussion

To the best of our knowledge, this is the largest hospital-based study performed to date examining the periprocedural and in-hospital outcomes in patients who underwent PCI in SVG from Pakistan. In our study population majority of patients presented with ACS, predominantly NSTEMI. More than 80% of our study population presented within 6-10 years since the last CABG was done. This study shows that SVG to OM is the most common graft and the body of the graft is the most common site of the lesion, which required intervention and the DES is the most common stent deployed in the SVG. In our study, population procedure success is very high however, 22 (13.2%) of patients developed periprocedural complications with predominantly slow flow and 7 (5%) of patients had in-hospital complications among which 4(2.4%) patients died during hospital stay.

ACS, predominantly NSTEMI was the most common presentation in our study population who required PCI in SVG and this finding is consistent with the study of Brilakis et al, in their study most of the patients presented with the ACS, predominantly NSTEMI for intervention in SVG [13].

In our study population, more than 80% of patients presented within 6-10 years of since last CABG done with graft failure. This finding is consistent with the Fitzgibbon GM et al study. In their study population, more than 50% of patients presented with graft failure within 10 years since the last CABG was done [5].

DES was the most commonly used in our study population this finding is consistent with the literature. Brilakis et al. and Mehilli et al. used DES in most of their study population [14,15]. Mosleh et al. and Ybarra et al. also used DES in most of their study population and it showed lesser adverse cardiac event as compared to BMS [16,17].

In our study population, the periprocedural complication was observed in 22 (13.4%) of patients with predominantly low flow in 13 (7.8%) of patients. In our study population, they developed slow flow in a lesser number as compared to Shakoor et al and Sharma et al. study populations. Shakoor et al. reported a slow flow of 11.5% [18] and Sharma et al. as high as 18% in their study population who underwent PCI in SVG [19].

There was only 4 (2.4%) mortality in our study population. All four patients died due to primarily underlying cardiovascular etiology. It is consistent with the results of the study conducted by Brilakis et al. and their study reported 5% mortality in their study population [20].

Our study is not free of limitations. It is a retrospective observational study, conducted in a single center and the sample size is small which may not represent the whole Pakistani population. There is no control group, so it is not possible to establish the association of PCI in SVG with mortality. There is no long-term follow-up. We suggest prospective randomized studies to predict the long-term outcomes of PCI in SVG in our region.

## Conclusions

PCI and CABG are complementary treatment modalities in patients with CAD. PCI in SVG lesions appears reasonable and safe with a decent periprocedural and in-hospital outcomes. Improvements in the expertise of interventionists and efficacy of antiplatelet, anticoagulation, and devices are expected to helper in more favorable outcomes for PCI in SVG.
